# Acylglycerol kinase promotes tumour growth and metastasis via activating the PI3K/AKT/GSK3β signalling pathway in renal cell carcinoma

**DOI:** 10.1186/s13045-019-0840-4

**Published:** 2020-01-03

**Authors:** Qian Zhu, Ai-Lin Zhong, Hao Hu, Jing-Jing Zhao, De-Sheng Weng, Yan Tang, Qiu-Zhong Pan, Zi-Qi Zhou, Meng-Jia Song, Jie-Ying Yang, Jun-Yi He, Yuan Liu, Min Li, Wan-Ming Hu, Chao-Pin Yang, Tong Xiang, Ming-Yuan Chen, Gang Ma, Ling Guo, Jian-Chuan Xia

**Affiliations:** 1 State Key Laboratory of Oncology in Southern China, Collaborative Innovation Center for Cancer Medicine, Guangzhou, China; 2 Department of Biotherapy, Sun Yat-Sen University Cancer Center, Guangzhou, 510060 People’s Republic of China; 3 Office of International Exchange and Cooperation, Guangzhou University of Chinese Medicine, Guangzhou, 510006 People’s Republic of China; 4 Department of Thoracic Surgery, Jiangxi Cancer Hospital, Nanchang, 330006 People’s Republic of China; 5 Department of Pathology, Sun Yat-Sen University Cancer Center, Guangzhou, 510060 People’s Republic of China; 6 Department of Experimental Research, Sun Yat-Sen University Cancer Center, Guangzhou, 510060 People’s Republic of China; 7 Department of Nasopharyngeal Carcinoma, Sun Yat-Sen University Cancer Center, Guangzhou, 510060 People’s Republic of China; 8 Department of Intensive Care Unit, Sun Yat-Sen University Cancer Center, Guangzhou, 510060 People’s Republic of China

**Keywords:** AGK, Renal cell carcinoma, Epithelial-mesenchymal transition, PI3K, AKT

## Abstract

**Background:**

Clinically, the median survival in patients with metastatic renal cell carcinoma (RCC) was only 6–12 months and a 5-year survival rate of less than 20%. Therefore, an in-depth study of the molecular mechanisms involved in RCC is of great significance for improving the survival of patients with advanced RCC. Acylglycerol kinase (AGK) is a newly discovered lipid kinase that has been reported to be a potent oncogene that may be involved in the regulation of malignant progression in a variety of tumours. However, the expression and biological characteristics of the AGK gene in RCC remain unclear.

**Methods:**

AGK expression was quantified by quantitative real-time PCR, Western blotting and immunohistochemistry in RCC cell lines and paired patient tissues. Kaplan-Meier method and Cox proportional hazards models were used to evaluate the prognostic value of AGK in human RCC tissue samples. Chi-squared test was performed to analyse the correlation between AGK expression and the clinicopathological features. Stable overexpression and knockdown of AGK in RCC cells was constructed with lentivirus. The oncogenic effects of AGK in human RCC progression were investigated using assays of colony formation, anchorage-independent growth, EdU assay, cell cycle analysis, wound-healing, trans-well analysis and xenograft tumour model. GSEA and KEGG analysis were conducted to detect the potential pathway of AGK involved in RCC. These results were further confirmed using the luciferase reporter assays, immunofluorescence and in vivo experiments.

**Results:**

AGK expression is significantly elevated in RCC and closely related to the malignant development and poor prognosis in RCC patients. By in vitro and in vivo experiments, AGK was shown to enhance the proliferation of RCC cells by promoting the transition from the G1 phase to the S phase in the cell cycle and to enhance the migration and invasion by promoting epithelial-mesenchymal transition. By activating the PI3K/AKT/GSK3β signalling pathway in RCC, AGK can increase nuclear accumulation of β-catenin, which further upregulated TCF/LEF transcription factor activity.

**Conclusions:**

AGK promotes the progression of RCC via activating the PI3K/AKT/GSK3β signalling pathway and might be a potential target for the further research of RCC.

## Introduction

Renal cell carcinoma (RCC) is the most common malignant cancer in the kidney [1]. Over the past 10 years, the incidence rate of RCC has increased at a rate of 2% per year [2]. It has been reported that the development of imaging techniques has led to an increase in early diagnosis [3]. However, 20–30% of patients still show evidence of distant metastases at the time of initial treatment [4]. Although surgery may be curative for early-stage RCC patients, deaths from RCC have not declined mainly because of recurrence and metastatic disease [5]. Because of the lack of an effective treatment, the median survival time of patients with RCC is only 6–12 months, and the 5-year survival rate is less than 20% [6]. Recent studies have shown that more than 90% of kidney cancer-related deaths are associated with the metastasis of RCC [7].

Acylglycerol kinase (AGK) is a lipid kinase that can catalyse the phosphorylation of acylglycerol to generate lysophosphatidic acid (LPA) [8, 9]. Bektas et al. confirmed that upregulation of LPA expression can increase its binding to epidermal growth factor receptor (EGFR), which changes the cytoskeletal structure, induces cell formation and promotes the metastasis of prostate cancer [10]. In addition, numerous studies have demonstrated that AGK is a powerful oncogene [11–17]. High expression of AGK can induce the expression of hypoxia-inducible factor 1a (HIF1a) and promote epithelial-mesenchymal transition (EMT) in cervical squamous carcinoma cells [11]. High expression of AGK is associated with lymph node metastasis and poor prognosis in patients with nasopharyngeal carcinoma [12]. AGK can activate the JAK2/STAT3 pathway and promote the malignant progression of oesophageal cancer [13]. Upregulation of AGK promotes angiogenesis of liver cancer by activating the NF-kB pathway and inhibits apoptosis of hepatoma cells [14]. High expression of AGK is associated with poor prognosis in patients with head and neck squamous cell carcinoma [15]. AGK can regulate the cell cycle in oral squamous cell carcinoma cells [16]. Wang et al. found that AGK can activate the PI3K/AKT signalling pathway and then inhibit cell cycle inhibitors in breast cancer [17]. However, the expression and biological characteristics of the AGK gene in RCC remain unclear.

The present study demonstrates that AGK expression in RCC is significantly higher than that in adjacent normal tissues. RCC patients with increased AGK expression experienced poorer prognosis and a higher risk of metastasis. By in vitro and in vivo assays, we found that AGK can promote the G1 phase to S phase transition and increase the proliferation of RCC cells. Meanwhile, AGK could induce RCC cell EMT and promote metastasis. Further studies confirmed that AGK upregulation could activate the PI3K/AKT/GSK3β pathway. Phosphorylation of GSK3β can inhibit the ubiquitination of β-catenin, leading to the accumulation of β-catenin in the cytoplasm and the upregulation various transcription factors. This study systematically explored the biological role of AGK in RCC and the molecular mechanism underlying its regulatory signalling pathway, which provides new targets for molecularly targeted therapy.

## Materials and methods

### Cell lines

Renal cancer cell lines (ACHN, Caki-2, A498, 786-O, Caki-1 and 769P) were purchased from the Cell Bank of the Chinese Academy of Sciences (Shanghai). Immortalised HK-2 renal tubular epithelial cells and the RCC cell line SK-RC-39 were gifted by Professor Dan Xie at the Sun Yat-sen University Cancer Center. The cells were cultured in a humidified incubator at 37 °C in 5% CO_2_ with RPMI 1640 medium containing 10% foetal bovine serum (FBS), 100 U/ml penicillin and 100 U/ml streptomycin. The cells were treated with the PI3K inhibitor LY294002 or transfected with a recombinant lentivirus carrying a human AGK overexpression plasmid, shRNA or the corresponding empty vectors (GenePharma, Shanghai, China).

### Patients

This study was conducted retrospectively in a cohort of 120 patients with primary RCC who were seen between January 2009 and August 2013 at the Sun Yat-sen University Cancer Center (Guangzhou, China). The tumour stage was defined according to the 7th edition of the UICC Staging System. All samples were collected with informed consent, and the Internal Review and the Ethics Boards of the Sun Yat-Sen University Cancer Center approved the ethical use of human subjects for this study.

### Real-time PCR

Total RNA was isolated from cell lines and freshly frozen tissues using Trizol reagent (Invitrogen). First-strand cDNA was synthesised using the PrimeScript™ RT Master Mix cDNA Synthesis Kit (TaKaRa) according to the manufacturer’s protocol. Quantitative PCR was conducted to detect gene mRNA expression using GoTaq qPCR Master Mix (Promega). The sequences of the primers used for amplifying AGK and GAPDH are listed in Additional file 1: Table S1.

### Western blotting

Western blotting was performed as previously described [12]. Briefly, protein samples (30 μg) were analysed by standard SDS/PAGE and transferred to PVDF membrane (Millipore). Membranes were blocked with 5% nonfat milk in Tris-buffered saline containing 0.1% Tween-20 (TBST) and revealed by blotting with the respective antibodies. GAPDH was used as an internal control. The primary antibodies used are shown in Additional file 1: Table S2.

### Immunohistochemical analysis and evaluation

The procedure was performed as previously described [18]. Primary antibodies against AGK, Ki-67 (Cell Signalling Technology) and β-catenin were diluted 1:100, 1:400 and 1:100, respectively. The score for each tissue was calculated by multiplying the staining value (0%, 0; 1–25%, 1; 26–50%, 2; 51–75%, 3; 76–100%, 4) by the percentage of stained cells (0, negative; 1, weak; 2, moderate; 3, intense). For Ki-67 evaluation, the percentage of positively stained cells among the total tumour cells was quantified. The scores were independently determined by two pathologists (Dr. Min Li and Wan-Ming Hu). The median IHC score was chosen as the cut-off value for defining high and low expression.

For histological evaluation (HE), mouse tumour-forming kidney and lung metastatic nodules were resected and fixed in 4% paraformaldehyde, followed by routine processing [19].

### CCK8 cell viability assay

Cells were seeded in 96-well plates at a density of 2 × 10^3^ cells/well. At each time point, 10 μl CCK8 solution was added directly to the test cells and incubated for 1 h at 37 °C. The absorbance was measured at 450 nm. The absorbance on days 1–5 was normalised to the absorbance on day 0, which was used as a control (100%). Each experiment was performed in triplicate.

### Colony formation assay

Cells (5 × 10^2^) were plated in 6-well plates and cultured for 10 days. The colonies were stained with 1% crystal violet stain for 30 s after fixation with 4% formaldehyde for 5 min. The colonies were counted, and the results are shown as the fold-change compared to the number of vector control cells.

### EdU labelling and immunofluorescence

Briefly, cells (4 × 10^5^) were incubated with EdU for 3 h at 37 °C. After washing three times with PBS, the cells were fixed with 4% paraformaldehyde for 15 min. The cells were then treated with click reaction buffer to visualise the anti-EDU-labelled cells according to the standard protocol. The percentage of EDU-positive cells was determined according to ten randomly chosen fields from three independent samples.

### Cell cycle analysis

Cells were harvested, washed twice with cold PBS, fixed in 75% ethanol and then incubated at 4 °C overnight. The fixed cells were centrifuged at 1000 g for 5 min and washed with cold PBS. Then, 20 μl RNase A was added to the cells and incubated for 30 min at 37 °C. The cells were centrifuged at 1000 g for 5 min and incubated with 400 μl propidium iodide (PI) staining solution at 4 °C in the dark.

### Wound-healing and transwell assays

For the wound-healing assay, cells were seeded in 6-well plates and allowed to grow to confluence. Wounds were created using a 10 μl pipette tip. The images of migration were obtained from the same field at 0 h and 24 h after wounding. For the Transwell migration assay, BD BioCoat Matrigel Invasion Chambers (Becton Dickinson Labware, Franklin Lakes, NJ) were used to perform the cell invasion assays. A total of 3 × 10^4^ cells in 200 μl of serum-free RPMI 1640 medium were placed in the upper compartment of a Transwell chamber. The lower chamber was filled with 500 μl RPMI 1640 medium containing 10% FBS. After 12 h of incubation, the cells on the lower surface were fixed, stained and counted. Five visual fields were randomly chosen, and the number of cells was counted under a microscope. For the Transwell invasion assay, BD BioCoat 9 Matrigel Invasion Chambers (Becton Dickinson Labware, Bedford, MA) were used, and the inserts were incubated for 24 h. All assays were performed in triplicate independently.

### Immunofluorescence

Cells were fixed for 15 min in 4% paraformaldehyde, washed with cold PBS twice, and then incubated with antibody at 4 °C overnight. After washing in PBS, the cells were incubated with the appropriate fluorochrome-conjugated secondary antibody for 1 h, and nuclear staining was performed with Hoechst dye for 10 min. Cells were observed under a fluorescence microscope.

### Human phospho-kinase antibody array

Proteome Profiler Human Phospho-Kinase Array Kit (ARY003B, R&D Systems, Inc. USA & Canada) was used to detect the relative levels of protein phosphorylation according to the manufacturer’s instruction. The spot signals were quantified using ImageJ software.

### Bioinformatics analysis

Gene expression profiles in renal cell carcinoma were downloaded from the Gene Expression Omnibus (GEO) database (GSE6344) (https://www.ncbi.nlm.nih.gov/geo). The expression of AGK was divided by the median of AGK mRNA expression. Gene Set Enrichment Analysis (GSEA) was performed using GSEA 2.0.9 (http://www.broadinstitute.org/gsea/) according to the guideline [20–22]. The significantly enriched pathways were identified from KEGG (Kyoto Encyclopedia of Genes and Genomes) pathway enrichment analysis. The pathway database was downloaded at (http://www.genome.jp/kegg/pathway.html). Fisher’s exact test and multiple comparison tests were used to calculate the *P* value and false positive rate (FDR) of each signalling pathway. According to the *P* value and FDR, we extracted the strong correlation signalling pathway of AGK gene in RCC.

### Luciferase reporter assay

Cells (5 × 10^5^) were seeded in 24-well plates and transfected with either a β-catenin-TCF/LEF-sensitive or -insensitive reporter vector (TOP FLASH/FOP FLASH, Promega) using Lipofectamine 2000 reagent in each well. After 24 h, the luciferase activity was measured using the Dual-Luciferase Reporter Assay System (Promega, CA, USA).

### Xenograft model

Female BALB/c nude mice (4 weeks of age) were purchased from the Shanghai Institute for Biological Sciences (Shanghai, China). For the kidney in situ tumour model, 5 × 10^6^ cells in 100 μl PBS were injected into the kidney using insulin syringes (Becton Dickinson). Tumour formation was observed by an IVIS 200 imaging system. For the lung metastasis model, 2 × 10^6^ cells in 100 μl PBS were injected into the tail vein using insulin syringes. The mice were sacrificed, and the number of metastatic nodules in each lung were counted 8 weeks after injection. For the subcutaneous tumour model, 5 × 10^6^ cells in 100 μl PBS were implanted under the right flanks of the mice. Tumour size and body weight were measured every 4 days. Six weeks later, the mice were sacrificed, and the tumour weights and volumes were calculated. This study protocol was approved by the Animal Care and Use Committee of the Sun Yat-Sen University Cancer Center, Sun Yat-Sen University.

### Statistical analysis

Statistical analyses were performed using SPSS version 19.0. A chi-squared test was performed to analyse the correlations between AGK expression and the clinicopathological features of the patients. Student’s *t* test was used to analyse the statistical significance of the differences between groups. The survival curves were determined using the Kaplan-Meier method and compared by the log-rank test. The overall survival (OS) of the patients following treatment was calculated according to the number of death events. The distant metastasis-free survival (DMFS) of the patients following treatment was calculated from the date of diagnosis to the date of the first distant metastasis at any site, death from any cause, or the date of the last follow-up visit. A Cox proportional hazards regression model was used for the multivariate survival analysis. *P* < 0.05 was considered statistically significant.

## Results

### AGK is overexpressed and correlated with poor survival in RCC

**Fig. 1:**
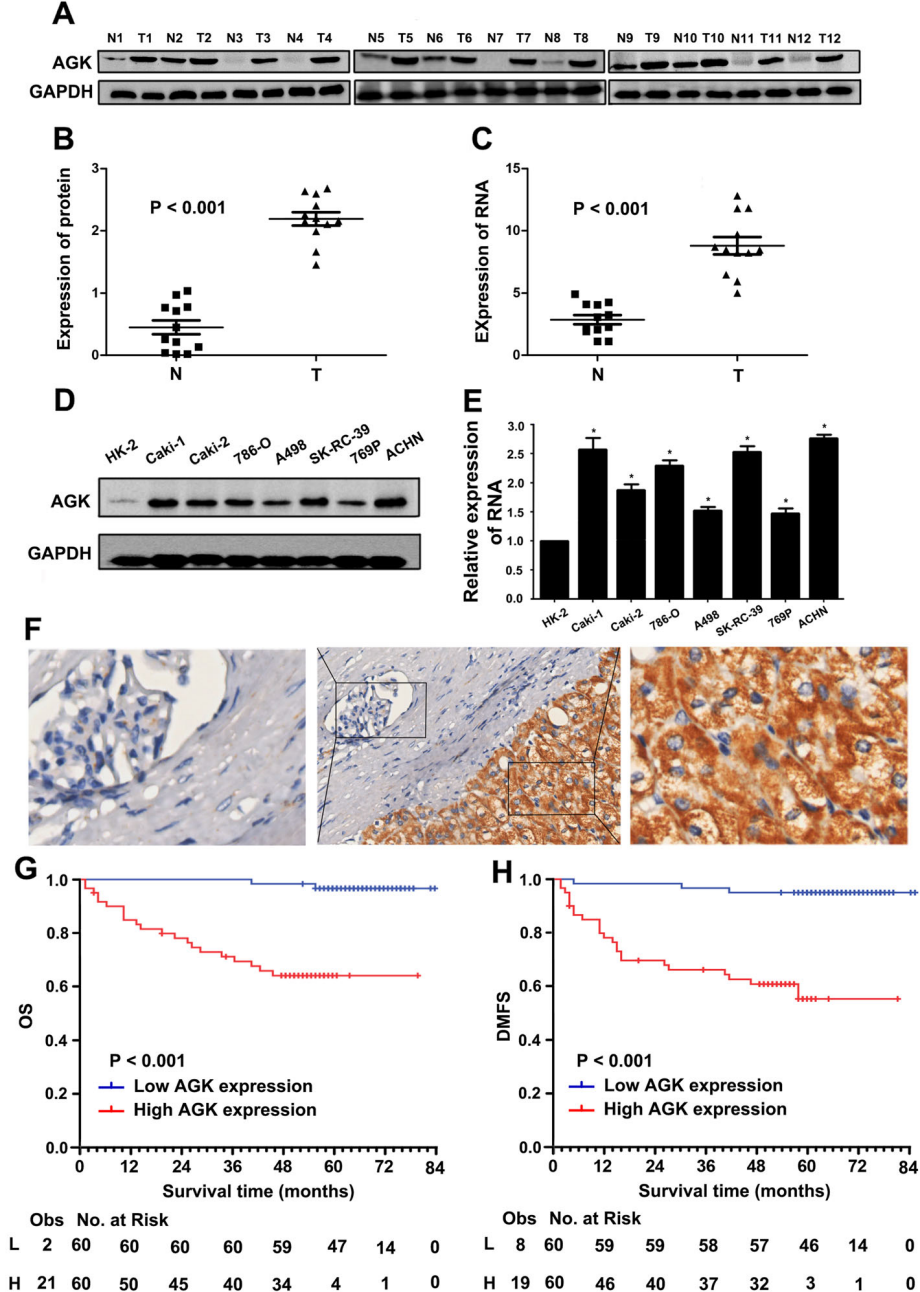
AGK is upregulated in RCC and is associated with poor prognosis in 120 RCC patients. **a** Representative images of AGK protein expression in 12 pairs of RCC tissues (T) and adjacent normal tissues (N). **b** Statistical analysis of the relative AGK protein levels in 12 pairs of RCC tumour samples and adjacent normal tissues. **c** Relative AGK RNA expression in 12 pairs of RCC tumour samples and adjacent normal tissues. **d** AGK protein and **e** mRNA expression were detected in a normal cell line (HK-2) and seven renal cell lines (Caki-1, Caki-2, 786O, A498, SK-RC-39, 769P and ACHN). GAPDH was used as a loading control. The error bars represent the standard deviation of the mean (SD) calculated from three experiments performed in parallel. *P* values were calculated using an independent Student’s *t* test. *P <  0.05 versus control. **f** Representative IHC images showing AGK expression in RCC tissue and adjacent normal tissue. **g** Kaplan-Meier analysis of overall survival (OS) and **h** distant metastasis-free survival (DMFS) according to AGK expression in 120 RCC patients

Western blotting and real-time PCR revealed that AGK protein and mRNA expression were upregulated in 12 human RCC tissues compared to that in the paired adjacent normal tissues (Fig. 1a–c). Consistently, AGK was shown to be elevated in seven RCC cell lines (Caki-1, Caki-2, 786-O, A498, SK-RC-39, 769P and ACHN) compared to its level in immortalised renal epithelial cell lines HK-2 (Fig. 1d, e). Furthermore, AGK expression was higher in the highly metastatic cell lines ACHN and Caki-1. IHC staining of paraffin-embedded archived biopsies further demonstrated that AGK was hardly observed in the adjacent normal tissues, while strong AGK expression was detected in the tumour tissues (Fig. 1f).

Correlation analysis showed that AGK expression was strongly associated with the clinical stage (*P* <  0.001), Fuhrman classification (*P* = 0.011), recurrence with metastasis (*P* <  0.001) and vital status (*P* <  0.001) (Table 1 and Additional file 1: Table S3). RCC patients with higher AGK expression experienced poorer 5-year OS (64% vs. 96%, *P* <  0.001, Fig. 1g) and 5-year DMFS (57% vs. 95%, *P* <  0.001, Fig. 1h) than patients with low AGK expression. Moreover, multivariate Cox regression analysis showed that the AGK protein expression level and clinical stage were independent prognostic indicators for RCC patients (Table 2). Importantly, patients with increased expression of AGK experienced an increased risk of death (HR 7.492, *P* = 0.008) and metastasis (HR 6.161, *P* = 0.004).

**Table 1:** Association between AGK expression and the clinicopathological features of RCC

Feature	No. of patients	AGK expression		*P*-value
		Low	High	
Gender				
Male	81 (67.5%)	41 (50.6%)	40 (49.4%)	1.000
Female	39 (32.5%)	19 (48.7%)	20 (51.3%)	
Age(years)				
< 50	68 (56.7%)	32 (47.1%)	36 (52.9%)	0.581
≥ 50	52 (43.3%)	28 (53.8%)	24 (46.2%)	
Family history of cancer				
Yes	10 (8.3%)	4 (40.0%)	6 (60.0%)	0.743
No	110 (91.7%)	56 (50.9%)	54 (49.1%)	
Clinical stage				
I-II	83 (69.2%)	51 (61.4%)	32 (38.6%)	**< 0**.**001**
III-IV	37 (30.8%)	9 (24.3%)	28 (75.7%)	
Pathological classification				
Renal clear cell carcinoma	116 (96.7%)	57 (49.1%)	59 (50.9%)	0.619
Others	4 (3.3%)	3 (75.0%)	1 (25.0%)	
Fuhrman classification				
I-II	96 (80.0%)	54 (56.3%)	42 (43.7%)	**0**.**011**
III-IV	24 (20.0%)	6 (25.0%)	18 (75.0%)	
Recurrence with metastasis				
Absent	93 (77.5%)	57 (61.3%)	36 (38.7%)	**< 0**.**001**
Present	27 (22.5%)	8 (11.1%)	19 (88.9%)	
Vital status				
Alive	97 (80.8%)	58 (59.8%)	39 (40.2%)	**< 0.001**
Dead	23 (19.2%)	2 (8.7%)	21 (91.3%)

**Table 2:** Univariate and multivariate Cox regression analysis of patient characteristics for overall survival and distant metastasis free survival among the 120 RCC patients

Covariant	OS		DMFS	
	HR (95% CI)	*P*	HR (95% CI)	*P*
Univariate analysis				
Gender (Female vs. Male)	0.581 (0.255–1.326)	0.197	0.641 (0.297–1.381)	0.256
Age (≤ 50 vs. > 50)	1.918 (0.789–4.663)	0.151	2.060 (0.901–4.707)	0.087
Family history of cancer (Y vs. N)	1.041 (0.244–4.442)	0.956	0.845 (0.200–3.566)	0.818
Clinical stage (I-II vs. III-IV)	34.361 (8.021–67.199)	**< 0**.**001**	19.200 (6.601–55.842)	**< 0**.**001**
Pathological classification (R vs. O)	1.232 (0.166–9.145)	0.838	1.211 (0.164–8.928)	0.851
Fuhrman classification (I-II vs. III-IV)	4.601 (2.023–10.464)	**< 0**.**001**	4.835 (2.265–10.318)	**< 0**.**001**
AGK expression (Low vs. High)	14.518 (3.369–62.556)	**< 0**.**001**	11.028 (3.285–37.021)	**< 0**.**001**
Multivariate analysis				
Clinical stage (I-II vs. III-IV)	22.264 (4.944–50.248)	**< 0**.**001**	11.727 (3.804–36.151)	**< 0**.**001**
Fuhrman classification (I-II vs. III-IV)	1.125 (0.483–2.620)	0.785	1.428 (0.648–3.146)	0.376
AGK expression (Low vs. High)	7.492 (1.701–32.998)	**0**.**008**	6.161 (1.801–21.070)	**0**.**004**

### AGK promotes the proliferation and tumourigenicity of RCC

**Fig. 2:**
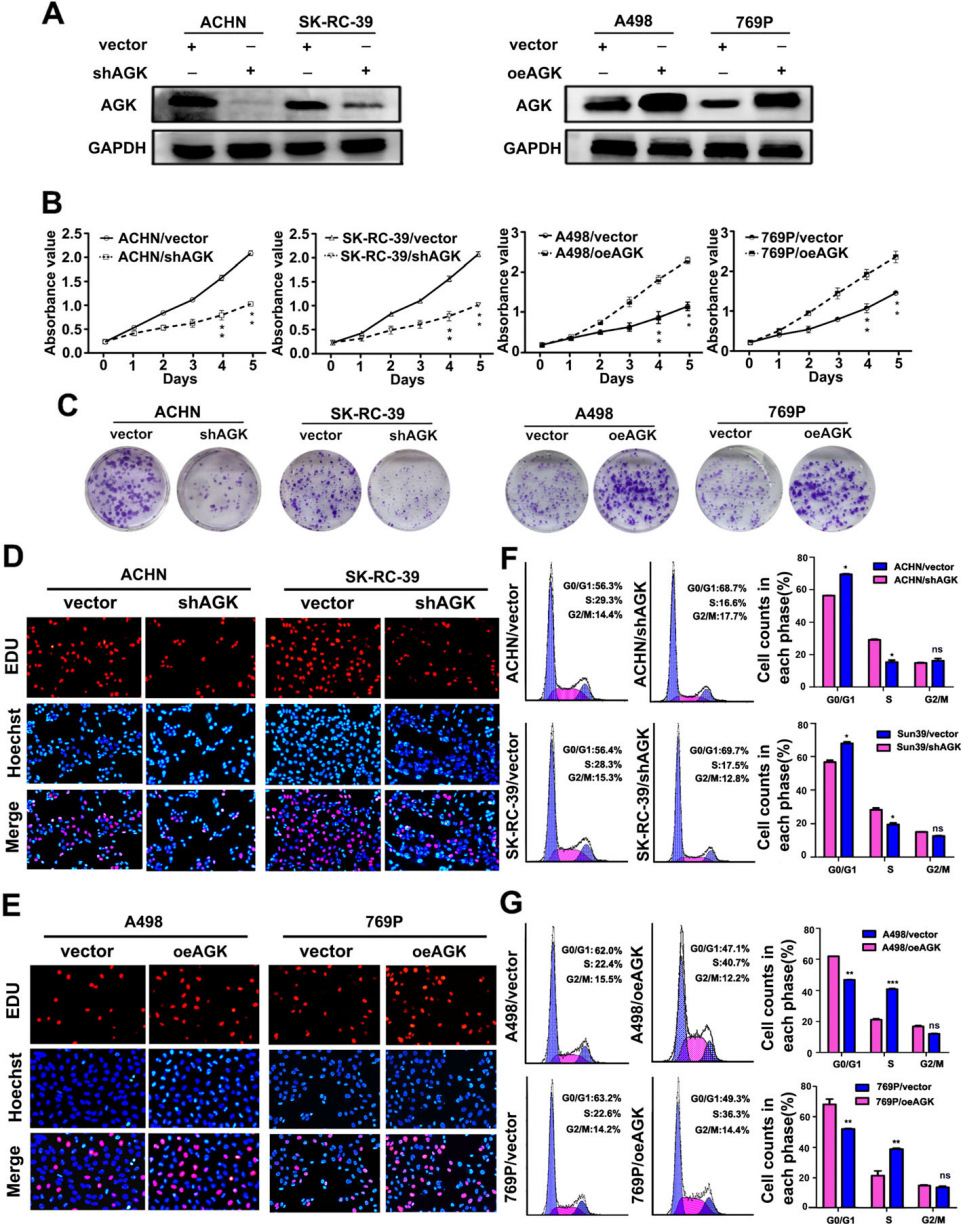
AGK promotes RCC cell proliferation. **a** Western blotting reveals that AGK was efficiently knocked down or overexpressed in corresponding cells. GAPDH was used as a loading control. **b** MTT assay and **c** colony formation assay showing the proliferation of the indicated RCC cells. Data are presented as the mean ± SD of three independent experiments. **d**, **e** Representative micrographs (left panel) of EDU incorporation detected in the indicated RCC cells. **f**, **g** Flow cytometric analysis (right panel) of the indicated RCC cells. The error bars represent the standard deviation of the mean (SD) calculated from three experiments performed in parallel. P-values were calculated using an independent Student’s t-test. *P <  0.05; **P <  0.01; ***P <  0.001; ns means non-significant

Since AGK expression was correlated with clinical stage in RCC, we then investigated the effect of AGK on the proliferation of RCC cells. We first knocked down AGK expression in RCC cell lines (ACHN and SK-RC-39) with relatively high expression of AGK, and we upregulated AGK expression in RCC cell lines (A498 and 769P) with relatively low expression of AGK (Fig. 2a). CCK8 and colony formation assays revealed that the proliferation rate of cells with increased AGK expression was significantly higher than that in the respective control cells, whereas the knockdown of AGK significantly reduced cell proliferation (Fig. 2b, c and Additional file 1: Figure S1).

EdU incorporation and flow cytometry assays showed that overexpressing AGK significantly increased the percentage of S phase cells, while the silencing of AGK reduced the percentage of S phase cells (Fig. 2d–g).

**Fig. 3:**
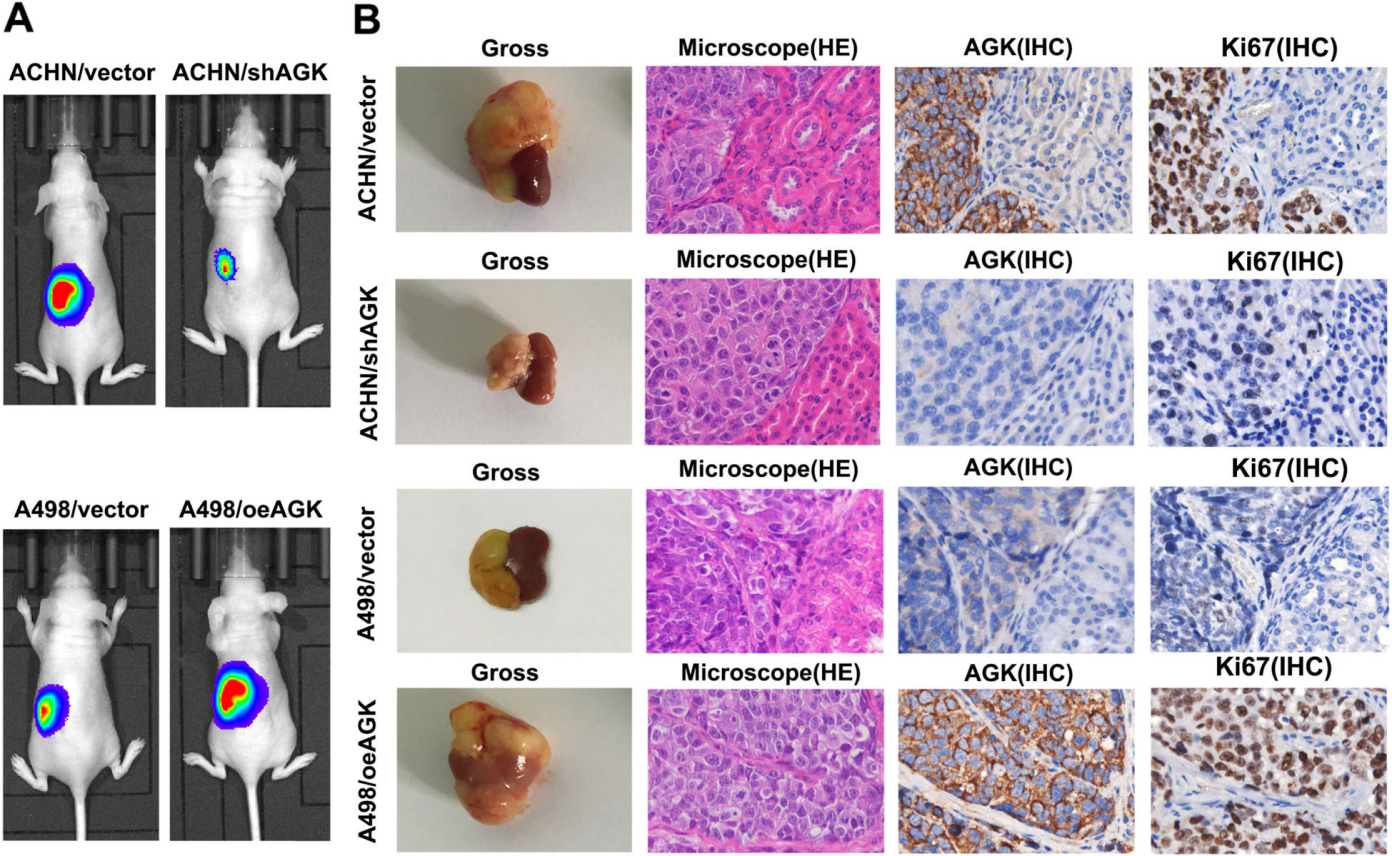
AGK promotes the tumourigenicity of RCC cells in vivo. **a** Representative bioluminescent images of tumours formed by the indicated cells. **b** Representative images of gross, H&E- and IHC-stained images of tumours in the tested mice

We next examined the effect of AGK on the tumourigenicity of RCC in vivo by using a kidney in situ tumour model. As shown in Fig. 3a, a remarkable increase in tumour growth was detected in A498/oeAGK tumours, whereas the growth of ACHN/shAGK cells was significantly reduced compared with that of the control ACHN/vector cells. Statistical analysis of tumour weights from all mice in each group further demonstrated the same results (Additional file 1: Figure S2A and B). H&E staining clearly revealed the adjacent normal tissues and cancer tissues. IHC was performed to detect the expression of AGK and Ki67. As shown in Fig. 3b, AGK was markedly highly expressed in tumour tissues compared with adjacent normal tissues. Furthermore, the level of Ki67 positively stained cells was higher in A498/oeAGK tumours, while the expression of Ki67 was remarkably reduced in ACHN/shAGK tumours. These results provide strong evidence that AGK plays a critical role in the proliferation of RCC cells.

### AGK significantly enhances cell migration and RCC metastasis

**Fig. 4:**
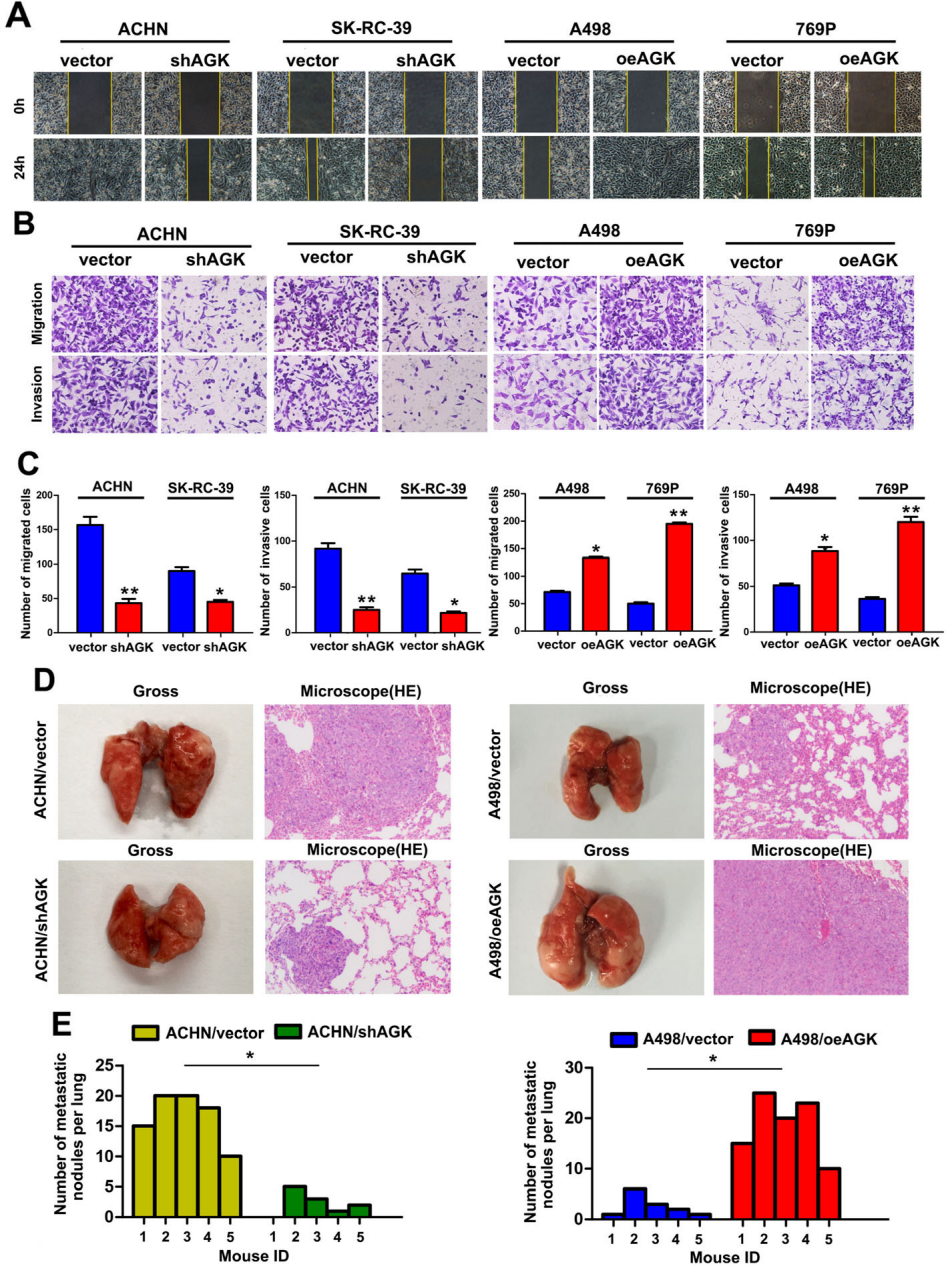
AGK significantly enhances cell migration and invasion in RCC. **a** Wound-healing assays showing the migration of the indicated RCC cells. **b** Transwell analysis showing the invasion of the indicated RCC cells. **c** Statistical analysis of the number of cells that passed through the chamber. **d** Representative images of lungs with metastatic nodules and H&E staining of lung metastatic tumours in the tested mice. **e** Statistical analysis of the number of metastatic nodules formed in the lungs of mice. *P <  0.05; **P <  0.01

Considering that AGK expression was significantly associated with recurrent metastasis, we further evaluated the effect of AGK on the metastasis of RCC cells. The wound-healing assay and the Transwell assay showed that overexpression of AGK significantly enhanced cell migration and invasion, while knockdown of AGK caused an apparent decrease in cell migration and invasion (Fig. 4a–c).

We next examined the effect of AGK on the metastasis of RCC in vivo by using a lung metastasis model. Four groups of 20 Balb/c nude mice were randomly assigned to receive tail vein injections of A498/oeAGK, A498/vector, ACHN/shAGK or ACHN/vector cells (*n* = 5/group). Eight weeks after injection, the weight of the lungs and the number of visible metastatic lymph nodes were determined and analysed. As shown in Fig. 4d, e, higher numbers of metastatic nodes were detected in tumours formed from A498/oeAGK cells, while tumours formed from ACHN/shAGK cells exhibited fewer metastatic nodes than tumours derived from vector cells. Meanwhile, a lower ratio of metastatic events was shown in the ACHN/shAGK group (80%, 4/5) than in the vector group (100%, 5/5). H&E staining of metastatic lesions in the lungs further confirmed the above results. Overall, these results demonstrated that AGK promotes RCC cell metastasis.

### AGK promotes epithelial-mesenchymal transition in RCC

**Fig. 5:**
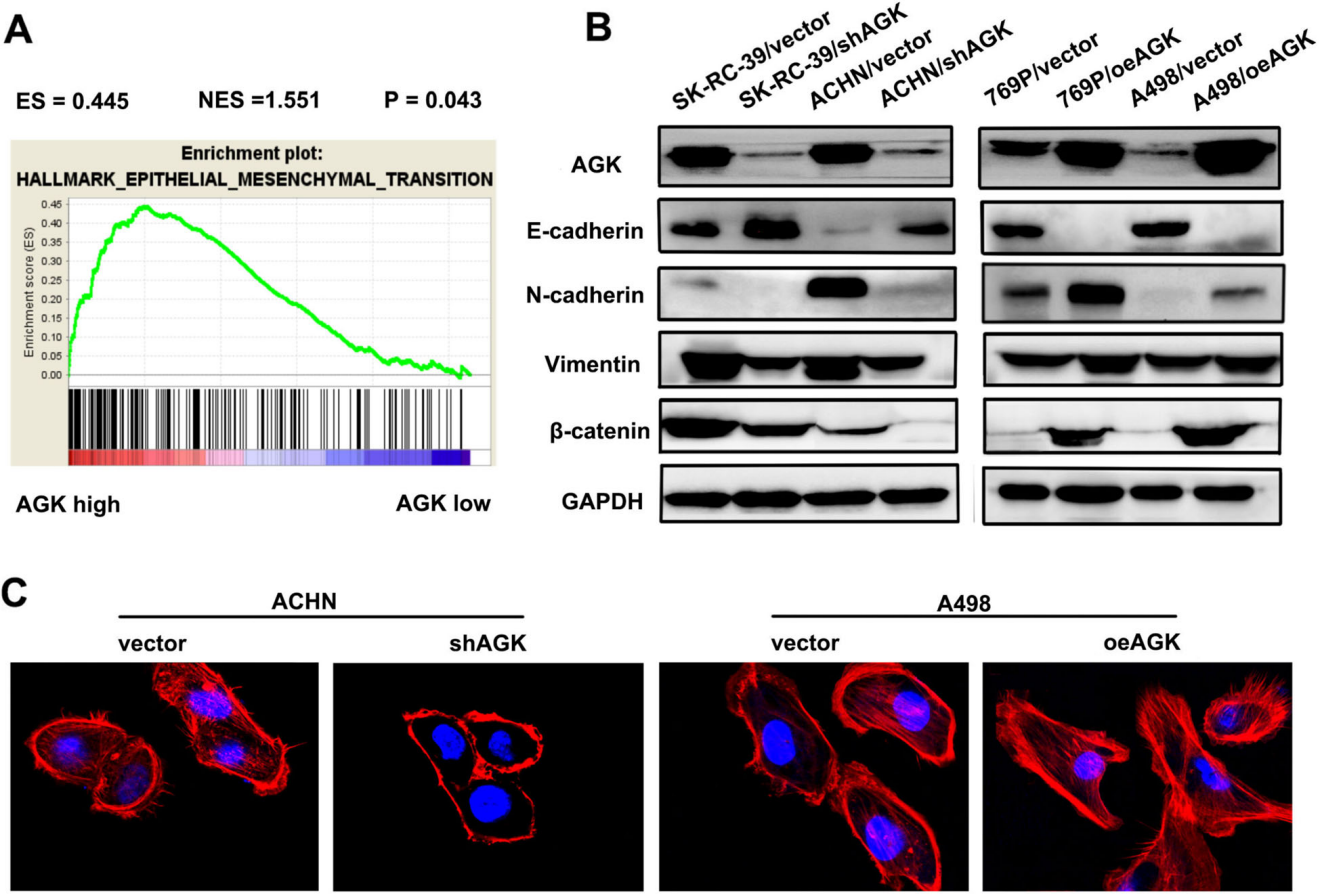
AGK induces epithelial-mesenchymal transition in RCC cells. **a** GSEA plot showing that AGK expression is positively correlated with EMT in RCC. **b** Western blot analysis of epithelial marker (E-cadherin) and mesenchymal marker (N-cadherin, vimentin and β-catenin) expression in the indicated cells. **c** Immunofluorescence assay of phalloidin (red) staining with DAPI counterstaining (blue) in the indicated cells. EMT refers to epithelial-mesenchymal transition

The GSEA plot revealed that AGK expression levels in RCC were positively correlated with EMT (*P* = 0.043, Fig. 5a). We therefore examined whether EMT was responsible for AGK-induced RCC cell metastasis. Western blotting demonstrated that the knockdown of AGK increased the expression of the epithelial marker E-cadherin and reduced the expression level of the mesenchymal markers N-cadherin, vimentin and β-catenin compared with those in the control group (Fig. 5b). Conversely, stable ectopic AGK-expressing A498 cells expressed a decreased level of E-cadherin and an increased level of mesenchymal markers. Moreover, phalloidin immunofluorescent staining indicated that AGK protein markedly altered the shapes of the RCC cells. We noticed that ACHN/shAGK cells displayed altered from a spindle-like shape to tight cell-to-cell adhesion as compared with the negative control cells. Many new spike-like protrusions and a more mesenchymal-type morphology were clearly observed at the edges of the AGK-overexpressing RCC cells (Fig. 5c). These results indicated that AGK promotes EMT in RCC.

### AGK promotes RCC proliferation and metastasis via the PI3K/AKT pathway

**Fig. 6:**
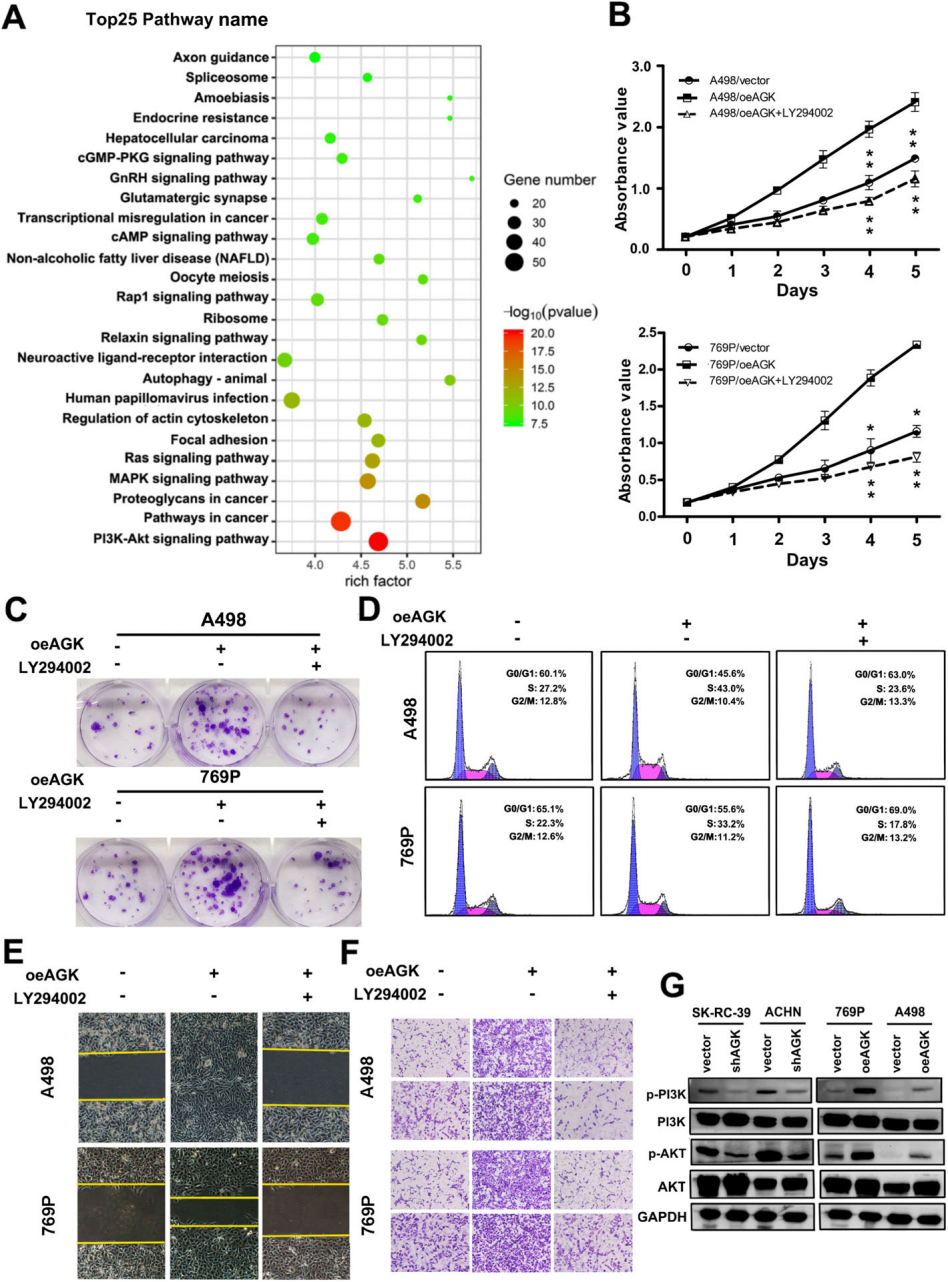
AGK stimulates the PI3K/AKT signalling pathway. **a** KEGG analysis was conducted to identify the pathways activated by AGK overexpression in RCC. **b** MTT assay, **c** colony formation assay and **d** flow cytometric analysis of the proliferation of the indicated RCC cells in the presence or absence of the PI3K inhibitor LY294002. Cell migration and invasion were measured by wound-healing (**e**) and Transwell assays (**f**) in the presence or absence of LY294002. **g** Western blotting analysis of the expression of p-AKT, p-PI3K, total AKT and total PI3K. GAPDH served as the loading control

Pathway enrichment analyses showed that the PI3K/AKT signalling pathway was significantly activated by AGK overexpression (Fig. 6a). Numerous studies have reported that the PI3K/AKT pathway plays an important role in metastasis through the regulation of EMT [23–25]. Therefore, the PI3K inhibitor LY294002 [26–29] was used to detect the effect of the PI3K/AKT pathway on AGK-induced RCC proliferation and metastasis. As shown in Fig. 6b–f, treatment with the PI3K inhibitor LY294002 in AGK-expressing cells markedly suppressed the proliferation, migration and invasion of RCC cells. Western blot analysis showed that AGK downregulation significantly reduced the expression of phospho-PI3K and phospho-AKT in both SK-RC-39 and ACHN cells, while the upregulation of AGK in 769P and A498 cells increased the expression of phospho-PI3K and phospho-AKT (Fig. 6g). These observations demonstrated that the PI3K/AKT signalling pathway is activated during the functional regulation of AGK-induced RCC.

### AGK activates GSK3β S9 phosphorylation sites, resulting in the inactivation of GSK3β

**Fig. 7:**
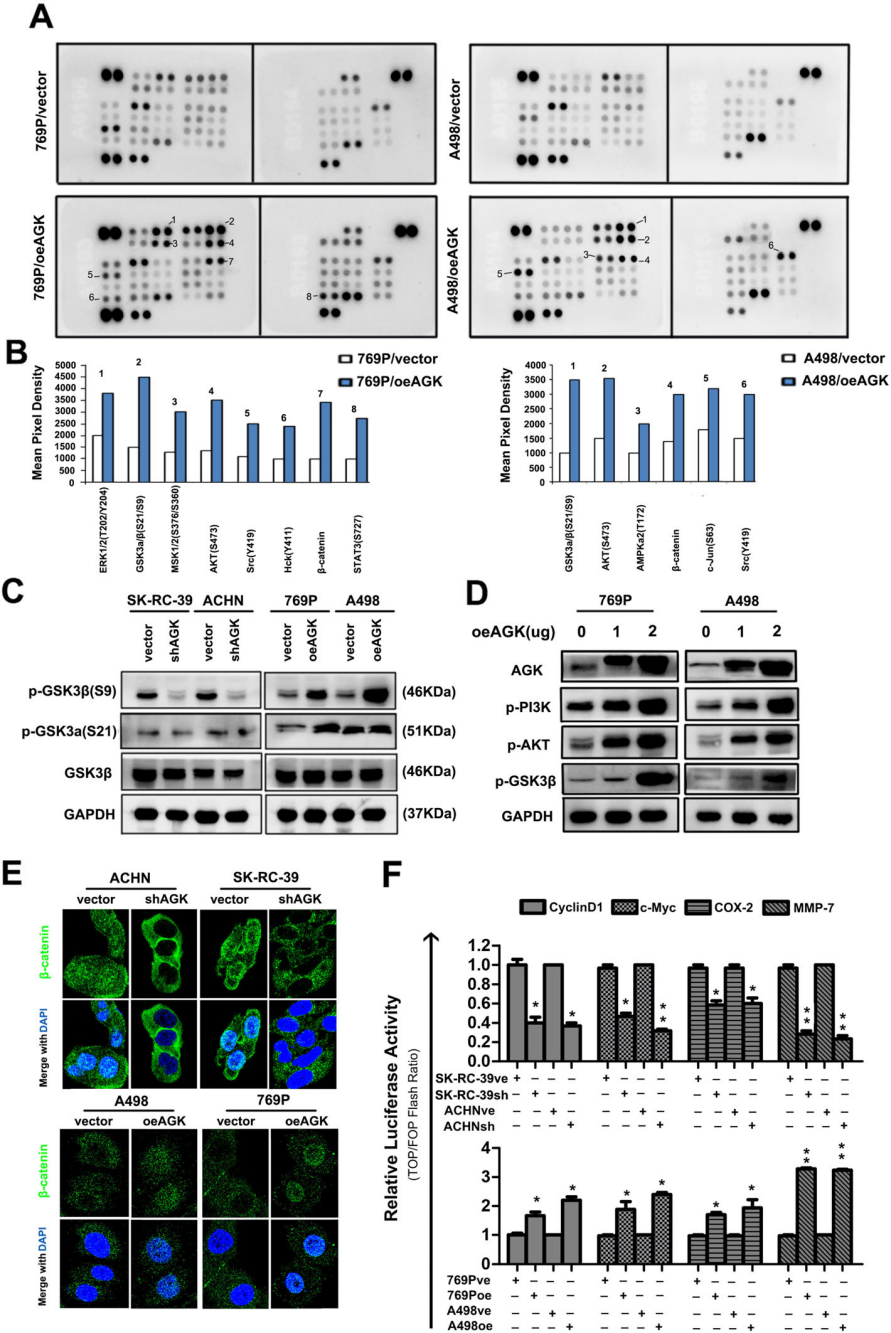
AGK activates the PI3K/AKT/GSK3β signalling pathway and β-catenin nuclear translocation in RCC cells. **a** Representative images of human phosphor-kinase array analysis. **b** Statistical analysis of the relative levels of the phosphorylation of 43 kinase phosphorylation sites in the indicated RCC cells. **c** The expression of p-GSK-3a and p-GSK-3β was detected by Western blot analysis. **d** Western blot analysis of the expression of p-AKT, p-PI3K and p-GSK-3β in cells transfected with a recombinant lentivirus carrying a human AGK overexpression plasmid. GAPDH served as the loading control. **e** The cellular location of β-catenin was examined by immunofluorescence staining. **f** Luciferase assay analysis of the transcriptional activity of TCF/LEF in the indicated cells. *P < 0.05; **P < 0.01

A human phosphor-kinase array was used to simultaneously detect the relative levels of phosphorylation of 43 kinase phosphorylation sites. The results show that upregulation of AGK in both 769P and A498 cells increased the phosphorylation of GSK3a/β (Fig. 7a, b). We further detected the expression of the two subtypes of GSK3, GSK3a and GSK3β, by specific antibodies. Western blotting demonstrated that AGK expression was associated with the level of GSK3β phosphorylation in RCC regardless of the level of phospho-GSK3a expression (Fig. 7c). Similarly, positive correlations were detected between AGK and phospho-PI3K, phospho-AKT and phospho-GSK3β (Fig. 7d).

### AGK promotes β-catenin translocation to the nucleus and upregulates TCF/LEF transcription factor activity

As GSK3β activity has been shown to be important for β-catenin degradation, the accumulation of β-catenin in the cytosol and its translocation from the cytosol into the nucleus was shown to result in the activation of TCF/LEF (T-cell factor/lymphoid enhancer factor) family transcription factors [30, 31]. Immuno-fluorescent staining indicated that AGK significantly increased nuclear β-catenin levels in RCC cells (Fig. 7e). The results of Western blotting further demonstrated overexpression of AGK increased nuclear β-catenin accumulation (Additional file 1: Figure S3). A luciferase reporter assay showed that the overexpression of AGK upregulated the activity of TCF/LEF family transcription factors, including cyclin D1, c-Myc, Cox2 and MMP-7, while the knockdown of AGK expression decreased the activity of cyclin D1, c-Myc, Cox2 and MMP-7 (Fig. 7f).

We concluded that if β-catenin is the downstream effector, then the inhibition of β-catenin should rescue AGK-induced malignant phenotypes. Therefore, we knocked down β-catenin expression by shRNA in cells with stable expression of AGK (Additional file 1: Figure S4A). As expected, the AGK-induced enhancement of cell viability was abolished by β-catenin shRNA (Additional file 1: Figure S4B-F). Furthermore, AGK-enhanced migration and invasion was partly attenuated by β-catenin shRNA (Additional file 1: Figure S4G). A dual-luciferase reporter assay demonstrated that decreased β-catenin expression in A498/oeAGK cells significantly inhibited the transcriptional activity of TCF/LEF family transcription factors (Additional file 1: Figure S4H and I). Similar to the results observed in the kidney in situ tumour model, A498/oeAGK tumours grew significantly faster than vector tumours, while tumours formed by A498/oeAGK/shβ-catenin cells grew more slowly (Additional file 1: Figure S4J-L). Moreover, β-catenin silencing in A498/oeAGK cells resulted in a significant decrease in the number of metastatic nodes (Additional file 1: Figure S4M and N). Overall, these findings suggest that AGK promotes β-catenin translocation to the nucleus, which further upregulates TCF/LEF transcription factor activity.

### AGK was positively correlated with β-catenin expression in human RCC samples

**Fig. 8:**
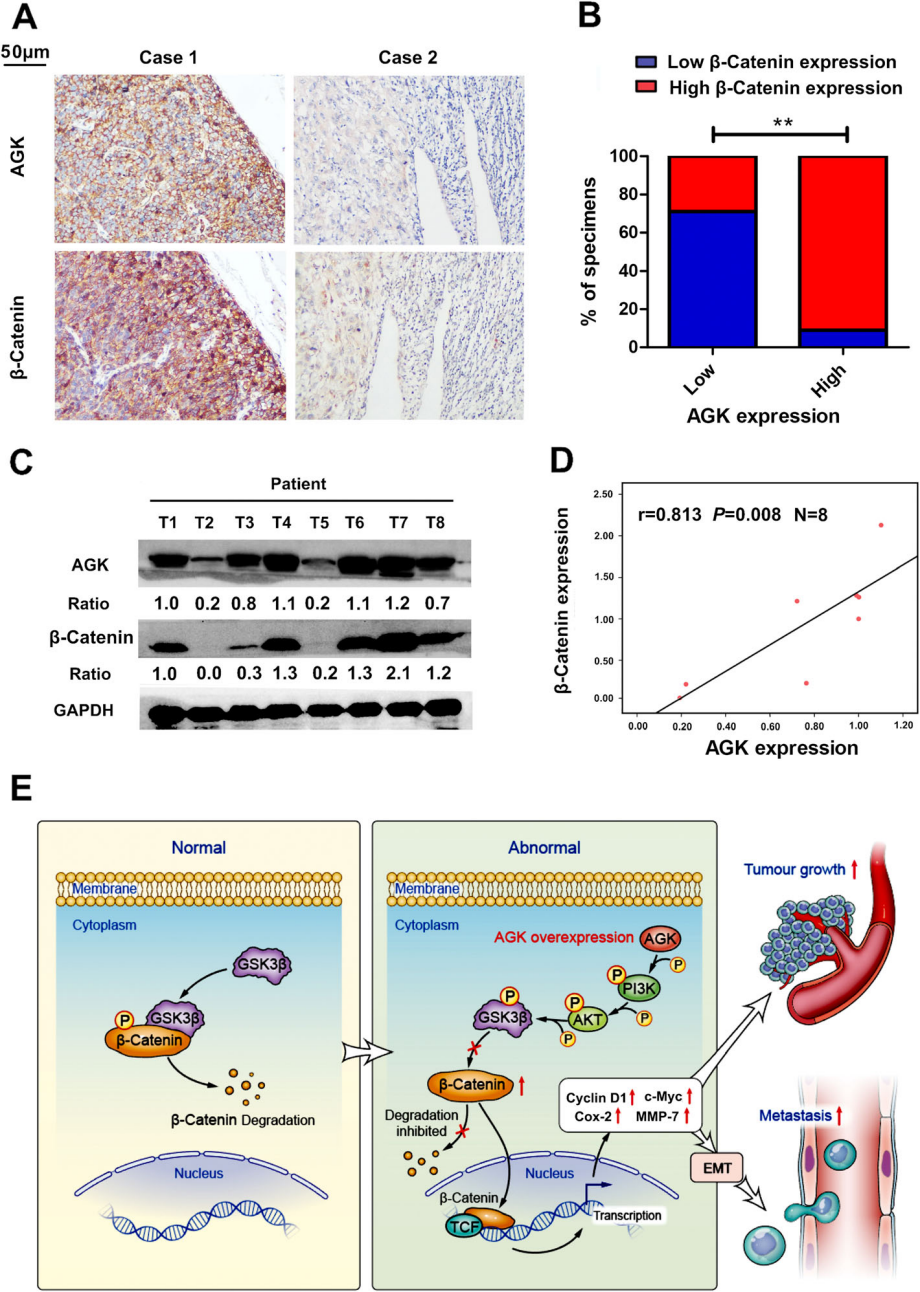
AGK expression is positively associated with β-catenin in RCC specimens. **a** Representative IHC staining images of β-catenin in tumour samples with high expression and low expression of AGK. **b** Statistical analysis of β-catenin and AGK expression detected by IHC staining. **c** Western blotting analysis of β-catenin and AGK protein expression in RCC tumour samples. **d** Statistical analysis of the association between β-catenin and AGK expression detected by Western blotting. **e** Schematic diagram depicting a proposed model for the major mechanism underlying the effects of AGK on the regulation of RCC proliferation and metastasis. **P < 0.01

IHC was performed to detect AGK and β-catenin expression in the same cohort of human RCC samples. The results showed that abnormal β-catenin expression was significantly increased in RCC tissues with high AGK expression compared to that in tissues with low AGK expression (Fig. 8a, b). Importantly, nuclear β-catenin expression was markedly increased in the AGK high expression group. Western blotting further demonstrated the positive association between AGK and β-catenin in RCC samples (*r* = 0.813, *P* = 0.008, Fig. 8c, d). Collectively, these findings implicate that AGK activates the PI3K/AKT/GSK3β signalling pathway, leading to the accumulation of β-catenin and the consequent upregulation of the activity of nuclear transcription factors in RCC (Fig. 8e).

## Discussion

The majority of kidney cancer-related deaths are associated with the metastasis of RCC [7]. Efforts have been increasingly made to discover the precise molecular mechanisms that drive this disease metastasis process to develop novel therapeutic targets for RCC. In this study, we demonstrated that AGK is upregulated and associated with several features of advanced disease. Further studies showed that RCC patients with increased levels of AGK experienced a higher risk of death and metastasis. The above data strongly suggest that AGK plays a role in the progression and metastasis of RCC. Therefore, further investigation of the mechanism is of great value for RCC treatment.

A previous study demonstrated that AGK can regulate the cell cycle of oral squamous cell carcinoma cells [16]. Wang et al. found that AGK can activate the PI3K/AKT signalling pathway and then inhibit cell cycle inhibitors in breast cancer [17]. This study demonstrated that AGK promotes the transition from the G1 phase to the S phase in the cell cycle and enhances the proliferation of RCC cells both in vitro and in vivo. Further investigation revealed a positive association between AGK and Ki67 expression. All results provide strong evidence that AGK is a proliferation-promoting oncogenic protein in RCC cells.

Several lines of evidence suggest that EMT promotes tumour metastasis in various cancers, including RCC [32–37]. Previous molecular analysis indicated that the high expression of AGK can induce the expression of HIF1a and promote EMT in cervical squamous carcinoma cells [11]. Our data show that AGK significantly enhances cell migration and promotes RCC metastasis. GSEA analysis detected a positive association between AGK and EMT in RCC. To confirm this result, we further evaluated the expression of the epithelial marker E-cadherin and mesenchymal markers N-cadherin, vimentin and β-catenin in the indicated RCC cell lines. Moreover, phalloidin immunofluorescent staining indicated that AGK protein markedly altered the cell shapes to a more mesenchymal phenotype compared with the shapes of negative control cells. These results demonstrated that the overexpression of AGK triggered an EMT-like phenotypic transition and promoted metastasis in RCC.

Numerous studies have reported that the PI3K/AKT/GSK3β/β-catenin pathway is aberrant and promotes proliferation, migration and invasion in a wide variety of cancers [38–42]. In this study, we found that the upregulation of AGK enhances the phosphorylation of multiple proteins, such as p-PI3K, p-AKT and p-GSK3β, in RCC. KEGG pathway enrichment analyses and comprehensive experiments confirmed that AGK promoted RCC proliferation and metastasis via the activation of the PI3K/AKT/GSK3β axis. It is well known that activation of GSK3β is required for the accumulation of β-catenin [43]. Upon phosphorylation of GSK3β at position Ser^9^ and the subsequent inactivation of GSK3β by p-AKT, cytosolic β-catenin degradation is attenuated, resulting in β-catenin accumulation in the cytoplasm and its translocation from the cytosol to the nucleus [30, 31]. As the present study has shown, along with increased AGK, increased nuclear accumulation of β-catenin is observed. Once in the nucleus, β-catenin acts as a transcriptional coactivator and activates the TCF/LEF (T cell factor/lymphoid enhancer factor) family of transcription factors [44, 45]. Studies have shown that β-catenin modifies cell-cycle activation [46] and cell–cell adhesion [47]. In this study, we found that AGK reduced GSK3β activity by phosphorylating Ser^9^, resulting in the nuclear accumulation of β-catenin, which further upregulated TCF/LEF transcription factor activity. In addition, a significantly positive association between AGK and β-catenin was detected in human RCC samples.

## Conclusion

In summary, our results indicate, for the first time, that AGK is a critical oncogenic factor and is associated with poor survival outcomes in RCC. Moreover, AGK promotes cell proliferation and metastasis through activation of the PI3K/AKT/GSK3β/β-catenin signalling pathway. These results might provide novel targets for the investigation of molecular treatments for RCC patients.

### Additional file

**Additional file 1: Table S1.** The sequences of the primers used for amplifying AGK and GAPDH. **Table S2.** The primary antibodies of Western blotting. **Table S3.** Association between the absolute IHC score of AGK expression and the clinicopathological features of RCC. **Figure S1.** AGK promotes RCC cell proliferation. **Figure S2.** AGK promotes the tumourigenicity of RCC cells in vivo. **Figure S3.** AGK altered nuclear translocation of β-catenin in RCC. **Figure S4.** β-catenin signalling is crucial for AGK-induced cell growth and invasion in RCC cells.

## Data Availability

The data are available to academic researchers upon request.

## Abbreviations

AGK: Acylglycerol kinase
DMFS: Distant metastasis-free survival
EGFR: Epidermal growth factor receptor
EMT: Epithelial-mesenchymal transition
FBS: Foetal bovine serum
HIF1a: Hypoxia-inducible factor 1a
LPA: Lysophosphatidic acid
OS: Overall survival
PI: Propidium iodide
RCC: Renal cell carcinoma
