# Correction: Acylglycerol kinase promotes tumour growth and metastasis via activating the PI3K/AKT/GSK3β signalling pathway in renal cell carcinoma

**DOI:** 10.1186/s13045-023-01505-6

**Published:** 2023-11-04

**Authors:** Qian Zhu, Ai-Lin Zhong, Hao Hu, Jing-Jing Zhao, De-Sheng Weng, Yan Tang, Qiu-Zhong Pan, Zi-Qi Zhou, Meng-Jia Song, Jie-Ying Yang, Jun-Yi He, Yuan Liu, Min Li, Wan-Ming Hu, Chao-Pin Yang, Tong Xiang, Ming-Yuan Chen, Gang Ma, Ling Guo, Jian-Chuan Xia

**Affiliations:** 1 State Key Laboratory of Oncology in Southern China, Collaborative Innovation Center for Cancer Medicine, Guangzhou, China; 2 Department of Biotherapy, Sun Yat-Sen University Cancer Center, Guangzhou, 510060 People’s Republic of China; 3 Office of International Exchange and Cooperation, Guangzhou University of Chinese Medicine, Guangzhou, 510006 People’s Republic of China; 4 Department of Thoracic Surgery, Jiangxi Cancer Hospital, Nanchang, 330006 People’s Republic of China; 5 Department of Pathology, Sun Yat-Sen University Cancer Center, Guangzhou, 510060 People’s Republic of China; 6 Department of Experimental Research, Sun Yat-Sen University Cancer Center, Guangzhou, 510060 People’s Republic of China; 7 Department of Nasopharyngeal Carcinoma, Sun Yat-Sen University Cancer Center, Guangzhou, 510060 People’s Republic of China; 8 Department of Intensive Care Unit, Sun Yat-Sen University Cancer Center, Guangzhou, 510060 People’s Republic of China

**Correction: Journal of Hematology & Oncology (2020) 13:2** 10.1186/s13045-019-0840-4

The original article [1] contains an erroneous bottom-right panel of Fig. 6F. The corrected sub-figure can be viewed ahead in this Correction article.

**Fig. 6:**
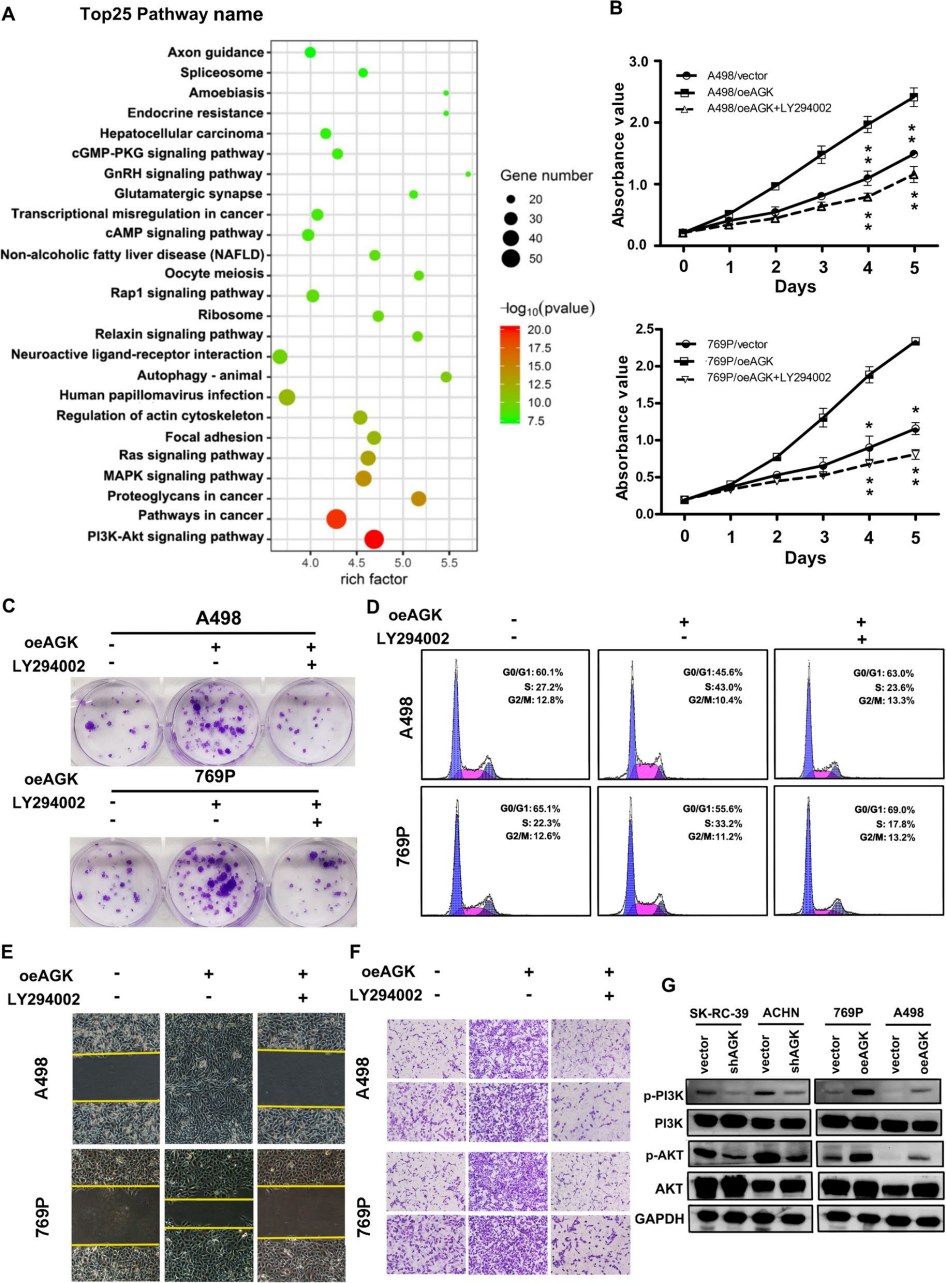
AGK stimulates the PI3K/AKT signalling pathway. **a** KEGG analysis was conducted to identify the pathways activated by AGK overexpression in RCC. **b** MTT assay, **c** colony formation assay and **d** flow cytometric analysis of the proliferation of the indicated RCC cells in the presence or absence of the PI3K inhibitor LY294002. Cell migration and invasion were measured by wound-healing (**e**) and Transwell assays (**f**) in the presence or absence of LY294002. **g** Western blotting analysis of the expression of p-AKT, p-PI3K, total AKT and total PI3K. GAPDH served as the loading control

