# Supplementary material for: Acylglycerol kinase promotes tumour growth and metastasis via activating the PI3K/AKT/GSK3β signalling pathway in renal cell carcinoma
Source: J Hematol Oncol. 2020 Jan 3;13:2. doi: 10.1186/s13045-019-0840-4 (PMC6942383; doi:10.1186/s13045-019-0840-4)
Supplement: Supplementary file 1 — Additional file 1: Table S1. The sequences of the primers used for amplifying AGK and GAPDH. Table S2. The primary antibodies of Western blotting. Table S3. Association between the absolute IHC score of AGK expression and the clinicopathological features of RCC. Figure S1. AGK promotes RCC cell proliferation. Figure S2. AGK promotes the tumourigenicity of RCC cells in vivo. Figure S3. AGK altered nuclear translocation of β-catenin in RCC. Figure S4. β-catenin signalling is crucial for AGK-induced cell growth and invasion in RCC cells. [file 13045_2019_840_MOESM1_ESM.docx]

**Supplementary Table 1** The sequences of the primers used for amplifying AGK and GAPDH

| Primer |  | 5’-3’ Sequence |
| --- | --- | --- |
| AGK | F | CGAAGGCTTGCGTCCTACTG |
|  | R | TGGTGGACAGCTGCACATCT |
| GAPDH | F | AAGGTCATCCCTGAGCTGAA |
|  | R | TGACAAAGTGGTCGTTGAGG |

**Supplementary Table 2** The primary antibodies of Western blotting

| Name of the antibody | Dilution ratio | Brand of the antibody |
| --- | --- | --- |
| anti-AGK | 1:1000 | Abcam |
| anti-E-cadherin | 1:500 | Cell Signaling Technology |
| anti-N-cadherin | 1:500 | Cell Signaling Technology |
| anti-Vimentin | 1:1000 | Cell Signaling Technology |
| anti-β-catenin | 1:1000 | Cell Signaling Technology |
| anti-AKT (pan) | 1:1000 | Cell Signaling Technology |
| anti-p-AKT | 1:1000 | Cell Signaling Technology |
| anti-GSK3β | 1:1000 | Cell Signaling Technology |
| anti-p-GSK3β | 1:1000 | Cell Signaling Technology |
| anti-p-GSK3a | 1:1000 | Cell Signaling Technology |
| anti-cyclinD1 | 1:1000 | Abcam |
| anti-c-Myc | 1:1000 | Abcam |
| anti-COX-2 | 1:1000 | Cell Signaling Technology |
| anti-MMP-7 | 1:1000 | Cell Signaling Technology |
| anti-GAPDH | 1:5000 | Abcam |

**Supplementary Table 3** Association between the absolute IHC score of AGK expression and the clinicopathological features of RCC

| No. of Patients  Feature | IHC score of AGK expression | | | | | | | | | P-value |
| --- | --- | --- | --- | --- | --- | --- | --- | --- | --- | --- |
|  | 0 | 1 | 2 | 3 | 4 | 6 | 8 | 9 | 12 |  |
| **Gender** |  |  |  |  |  |  |  |  |  |  |
| Male | 9 | 10 | 9 | 9 | 4 | 9 | 13 | 14 | 4 | 0.303 |
| Female | 0 | 3 | 5 | 6 | 5 | 6 | 5 | 6 | 3 |  |
| **Age(years)** |  |  |  |  |  |  |  |  |  |  |
| $<$ 50 | 2 | 8 | 9 | 7 | 6 | 9 | 10 | 11 | 6 | 0.218 |
| $\geq$ 50 | 7 | 5 | 5 | 8 | 3 | 6 | 8 | 9 | 1 |  |
| **Family history of cancer** |  |  |  |  |  |  |  |  |  |  |
| Yes | 0 | 0 | 2 | 2 | 0 | 2 | 1 | 2 | 1 | 0.409 |
| No | 9 | 13 | 12 | 13 | 9 | 13 | 17 | 18 | 6 |  |
| **Clinical Stage** |  |  |  |  |  |  |  |  |  |  |
| I-II | 9 | 13 | 14 | 13 | 2 | 11 | 11 | 10 | 0 | **<0.001** |
| III-IV | 0 | 0 | 0 | 2 | 7 | 4 | 7 | 10 | 7 |  |
| **Pathological classification** |  |  |  |  |  |  |  |  |  |  |
| RCC | 11 | 15 | 14 | 9 | 8 | 21 | 15 | 16 | 7 | 0.113 |
| Others | 1 | 1 | 1 | 0 | 0 | 1 | 0 | 0 | 0 |  |
| **Fuhrman classification** |  |  |  |  |  |  |  |  |  |  |
| I-II | 9 | 13 | 13 | 12 | 7 | 12 | 13 | 15 | 2 | **<0.001** |
| III-IV | 0 | 0 | 1 | 3 | 2 | 3 | 5 | 5 | 5 |  |
| **Recurrence with Metastasis** |  |  |  |  |  |  |  |  |  |  |
| Absent | 9 | 13 | 13 | 15 | 7 | 11 | 14 | 11 | 0 | **<0.001** |
| Present | 0 | 0 | 1 | 2 | 5 | 1 | 2 | 9 | 7 |  |
| **Vital status** |  |  |  |  |  |  |  |  |  |  |
| Alive | 9 | 13 | 14 | 15 | 7 | 11 | 14 | 12 | 2 | **<0.001** |
| Dead | 0 | 0 | 0 | 0 | 2 | 4 | 4 | 8 | 5 |  |

**Supplementary Figure 1: AGK promotes RCC cell proliferation. (A)** Statistic analysis result of colony formation in the indicated AGK KD cells and **(B)** AGK OE cells. *P < 0.05; **P < 0.01.

**
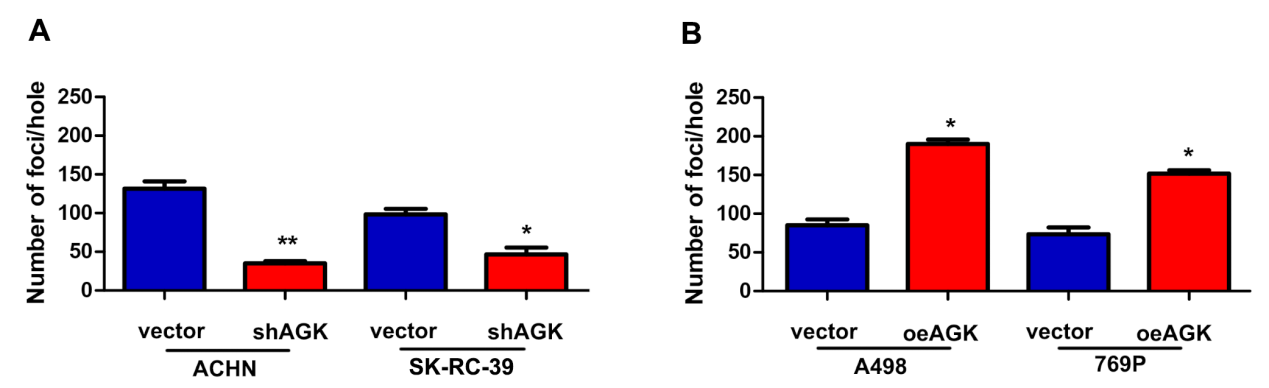
**

**Supplementary Figure 2: AGK promotes the tumourigenicity of RCC cells *in vivo*. (A)** Representative bioluminescent images of tumours formed by the indicated cells in each group. **(B)** Tumour weights from all mice in each group. ***P < 0.001.

**
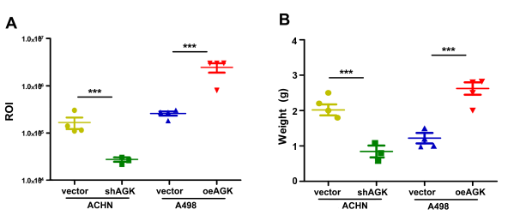
**

**Supplementary Figure 3:** AGK altered nuclear translocation of β-catenin in RCC. Western blotting analysis of β-catenin in nuclear and cytosolic extracts of RCC cells. The cytosolic or nuclear levels of β-catenin were normalized to GAPDH or P84 respectively.

**
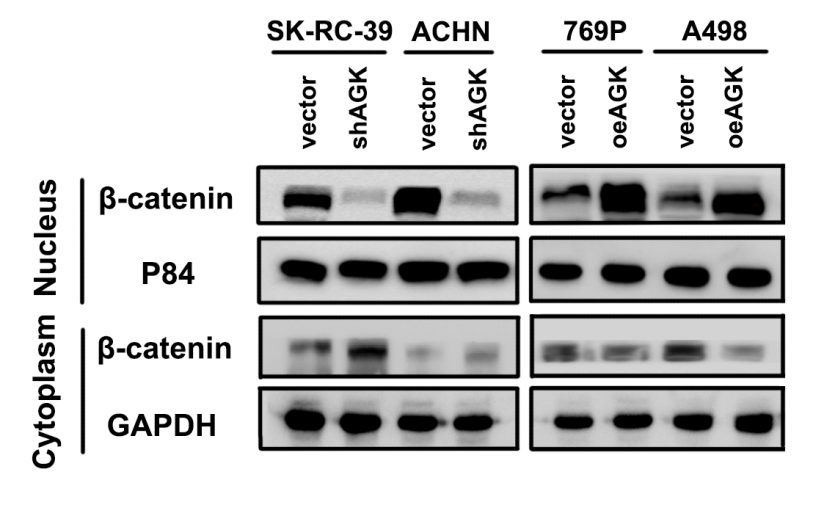
**

**Supplementary Figure 4: β-catenin signalling is crucial for AGK-induced cell growth and invasion in RCC cells. (A)** Western blot analysis of the effect of knocking down the expression of β-catenin. **(B)** MTT assay and **(C, D)** colony formation assay showing the proliferation of the indicated RCC cells. **(E, F)** Flow cytometric analysis (right panel) of the indicated RCC cells. **(G)** Wound-healing and Transwell assays of the migration and invasion of the indicated RCC cells. **(H)** Luciferase assay analysis of the transcriptional activity of TCF/LEF family members in the indicated cells. **(I)** Cyclin D1, c-Myc, Cox2, and MMP-7 expression detected by Western blot analysis. **(J-L)** Representative images of gross, H&E- and IHC-stained images of tumours in the tested mice. **(M, N)** Representative images of lungs with metastatic nodules and H&E staining of lung metastatic tumours in the tested mice.

**
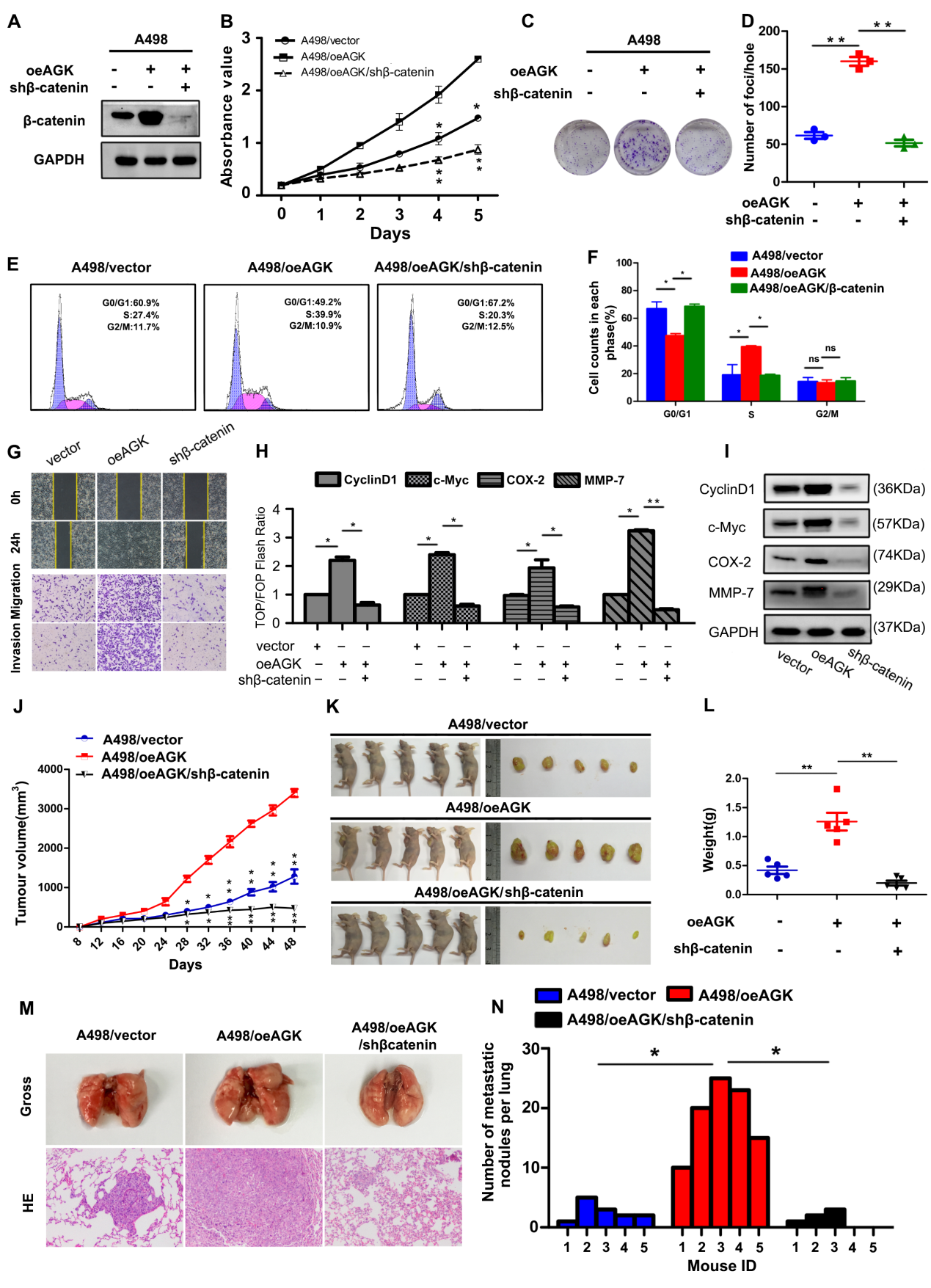
**
